# The attitudes, knowledge and confidence of healthcare professionals about cannabis-based products

**DOI:** 10.1186/s42238-024-00242-y

**Published:** 2024-07-24

**Authors:** Emilio Russo, Paula Martinez Agredano, Peter Flachenecker, Charlotte Lawthom, Duncan Munro, Chandni Hindocha, Makarand Bagul, Eugen Trinka

**Affiliations:** 1 Science of Health Department, Magna Grecia University, Catanzaro, Italy; 2 University Hospital Reina Sofia, Córdoba, Spain; 3 Neurological Rehabilitation Centre Quellenhof, Bad Wildbad, Germany; 4 University of Swansea, Swansea, UK; 5 Lumanity, London, UK; 6 GW Pharmaceuticals (Part of Jazz Pharmaceuticals), Cambridge, UK; 7 Department of Neurology, Christian-Doppler University Hospital, Paracelsus Medical University, Centre for Cognitive Neuroscience Salzburg, Member of the European Reference Network, EpiCARE, Salzburg, Austria; 8 Neuroscience Institute, Christian-Doppler Medical Centre, Paracelsus Medical University, Centre for Cognitive Neuroscience Salzburg, Salzburg, Austria; 9 Karl Landsteiner Institute of Neurorehabilitation and Space Neurology, Salzburg, Austria

**Keywords:** Healthcare professional education, Survey, Cannabis, Cannabinoids, Cannabis-based product, Cannabis-based medicine

## Abstract

**Background:**

Use of cannabis-based products is becoming more frequent, and it is important that healthcare professionals are informed and confident about them when making evidence-based decisions about their use. This study aimed to gain an international perspective on the attitudes, knowledge, and confidence of healthcare professionals about cannabis-based products.

**Methods:**

An online questionnaire regarding these products was completed by 1580 healthcare professionals (neurologists, psychiatrists, general practitioners, pharmacists and nurses) from 16 countries across Asia, Europe, Oceania, South America, and the Middle East.

**Results:**

Respondents expressed a high level of interest in cannabis-based products (median score 9 out of 10) and reported that they felt knowledgeable about them (median score 6 out of 7). They reported a high level of confidence when providing patients with information on cannabis-based products, returning median scores of 6 and 5 out of 7 for their legality and regulations, and their benefits and risks, respectively. Despite this, healthcare professionals sought further information on cannabis-based products across areas including legality, neurobiology, and scientific evidence. Finally, 59% (n = 930) of respondents considered robust clinical trial evidence as the most important factor to ensure patient safety in the context of these products. Few nominally significant differences emerged between healthcare professionals from different specialities or regions.

**Conclusion:**

In conclusion, this large survey of attitudes held by healthcare professionals towards cannabis-based products revealed a high level of interest and a demand for more information. Limitations of this study include potential sample bias and limited external validity.

**Supplementary Information:**

The online version contains supplementary material available at 10.1186/s42238-024-00242-y.

## Introduction

Cannabis refers to the *Cannabis sativa* L plant and its products (Morales et al. 2017), and although cannabis has many applications, its potential use in healthcare has attracted substantial research interest in recent years. Cannabis contains large amounts of phytocannabinoids, a class of over 120 aromatic hydrocarbons that are of interest to medical research due to their therapeutic potential (Morales et al. 2017), including delta-9-tetrahydrocannabinol (THC), cannabidiol (CBD), cannabigerol (CBG), and cannabidivarin (CBDV) (Morales et al. 2017). Some of the health effects caused by cannabinoids result from their interaction with the endocannabinoid system, a highly complex regulatory system that governs a multitude of biological processes (Ligresti et al. 2016; Hillard 2018; Marzo et al. 1998; Devane et al. 1992). Dysregulation of the endocannabinoid system is known to play a role in various neuropsychiatric disorders, which partly explains why the therapeutic potential of cannabinoids is of increasing scientific interest (Cristino et al. 2020; Morano et al. 2020; Stasiulewicz et al. 2020). Cannabinoids can also interact, sometimes exclusively, with molecular targets outside of the endocannabinoid system, allowing them to affect more physiological processes than previously thought (Morales et al. 2017; Cristino et al. 2020).

Growing curiosity about cannabis has coincided with an increase in the number, variety, and availability of cannabis-based products. ‘Medical cannabis’ can refer to a group of non-regulatory approved cannabis-based products that can be legally prescribed by physicians in certain countries (Pratt et al. 2019; Bettiol et al. 2018). Additionally, cannabis-based products containing CBD that are sold in shops or online are becoming more widely available to the general public (Bhamra et al. 2021). Neither of these product categories have been reviewed or approved by medicines regulators, such as the European Medicines Agency (EMA) or the United States Food and Drug Administration (FDA), they do not have high-quality evidence supporting their efficacy or safety profile, and their cannabinoid content is often inconsistent with what is reported on their packaging (Bhamra et al. 2021; Bonn-Miller et al. 2017; Liebling et al. 2020). Regulations governing the use and availability of cannabis-based products vary by country (McGregor et al. 2020; Schlag et al. 2020). As the availability of these different types of cannabis-based products increase globally, it is important that healthcare professionals (HCPs) are aware of these products, their differences, and the quality of evidence that exists regarding their proposed health effects.

Only a small number of cannabis-based medicines have been approved for therapeutic use in specific patient populations by medicines regulators in the UK and Europe (Centre and for Drugs and Drug Addiction 2018). Approved therapeutic indications in these regions include the treatment of seizures associated with Lennox–Gastaut syndrome (LGS), Dravet syndrome (DS), or tuberous sclerosis complex (TSC) in patients aged ≥ 2 years of age (Epidyolex®, in conjunction with clobazam for management of LGS or DS), symptomatic relief of spasticity in patients with multiple sclerosis who have not responded adequately to other therapy (Sativex®), chemotherapy-associated nausea and vomiting after previous treatments have failed (Marinol®, Syndros®, Cesamet®, and Canemes®), and anorexia associated with acquired immune deficiency syndrome (AIDS, Marinol® and Syndros®) (European Monitoring Centre for Drugs and Drug Addiction 2018; European Medicines Agency 2021). These approvals were based on randomized, double-blind clinical trial data in support of their quality, efficacy and safety (European Monitoring Centre for Drugs and Drug Addiction 2018; European Medicines Agency 2016). This quality of data does not exist for non-regulatory approved cannabis-based products, including medical cannabis and CBD products sold directly to consumers, which may pose a challenge to HCPs when answering questions from patients on a wide range of topics around cannabis-based products (Pratt et al. 2019; Chesney et al. 2020). The growing amount of misinformation being published about the health effects of cannabis-based products, often online and through social media (Allem et al. 2020), (Kruger et al. 2020), may also affect the ability of HCPs to confidently address questions from their patients.

As such, there is a need to better understand the current attitudes, knowledge, and confidence that HCPs have about the use of cannabis-based products in healthcare. Given education on cannabinoid science is not routine during most medical training courses, providing further education for HCPs on clinically relevant topics in cannabinoid science may be beneficial. Additional education may enable HCPs to have better informed conversations with their patients about the use of cannabis-based products. Existing studies surveying the attitudes, knowledge, and confidence of HCPs about cannabis-based products in healthcare have focused on a relatively small number of HCPs from specific medical specialities and individual countries or regions (Philpot et al. 2019; Szaflarski et al. 2020; Balneaves et al. 2018; Boehnke et al. 2021; Elliott et al. 2020; Arnfinsen and Kisa 2020; Jacobs et al. 2019; Kondrad and Reid 2013; Chan 2017). Furthermore, there is a relative lack of information on the attitudes of HCPs from outside North America towards cannabis-based products, in particular HCPs from Europe (Hordowicz et al. 2021; Gardiner et al. 2019).

The aim of this study was to gain an international perspective on the attitudes, knowledge, and confidence of healthcare professionals about cannabis-based products and medicines. To achieve this, HCPs from multiple medical specialities across Asia, Europe, Oceania, South America, and the Middle East were surveyed using an online questionnaire.

## Methods

### Survey design

The online questionnaire was designed by GW Pharmaceuticals (part of Jazz Pharmaceuticals), in partnership with the healthcare advisory company, Cello Health (London, UK). The survey was composed of 40 questions, including those used for screening purposes.

The questionnaire was developed in collaboration between GW Pharmaceuticals (part of Jazz Pharmaceuticals, Cambridge UK) and Cello Health according to an initial specification laid out by GW Pharmaceuticals when they initially requested Cello Health to conduct this work which was to cover the following areas of interest:

- HCPs attitudes towards use of cannabis-based medicines
- Extent to which HCPs differentiate between pharmaceutical companies and cannabis companies
- Baseline understanding of neurobiology of cannabinoids
- Perceptions of the level and quality of evidence around efficacy and safety of cannabinoids to treat different conditions
- Understanding of regulations around the use of cannabis-based medicines
- The perceived place of non-regulatory-approved cannabis-based products in patient care
- The propensity of patients to ask about non-licensed cannabis-based medicines or CBD food products and how well-prepared HCPs feel to discuss these with patients
- Understand what HCPs feel the most pressing education needs are

The survey was then designed to meet this broad specification. It was also made clear in this initial specification that this needed to be agnostic of specific GW Pharmaceutical products and therapy areas and should not give undue prominence to GW Pharmaceutical brands, which are mentioned only sparingly in the final questionnaire and alongside competitor products.

The same survey was provided to all respondents, following translation to the native language of the respondent. The translation process had two phases. The survey was first translated from English into each local language using a professional translation agency, Global Lexicon. The translated surveys were then independently checked by a freelance translator, who was a native speaker in that language, and any required amendments were made by Global Lexicon. In cases where questions were not required to be answered by respondents from certain medical specialities, this is stated in the manuscript. The survey aimed to gather information on various topics around cannabis-based products, including baseline awareness and perceptions of cannabis-based products; numbers of prescriptions and recommendations of cannabis-based products by HCPs; levels of understanding and knowledge around the medical use of cannabinoids; understanding of the legal regulations around cannabis-based products; and confidence in discussing cannabis-based products with patients. To minimize bias, respondents were not aware that the survey data were being collected on behalf of GW Pharmaceuticals (part of Jazz Pharmaceuticals, Cambridge UK).

The panel was compiled by GRG Health over a period of years by inviting individual HCPs to join this panel for the specific purpose of market research. All participating HCPs had previously opted in to receive requests to participate in market research surveys and provided their written consent to participate in this survey.

### Market research and ethics guidelines

This manuscript reports the results from a market research survey, which was carried out in accordance with guidelines set by the European Pharmaceutical Market Research Association (EphMRA) and the British Healthcare Business Intelligence Association (BHBIA). These guidelines state that approval from an ethics committee is not required to undertake market research. In line with BHBIA guidance, respondents were informed that the survey was being carried out by Cello Health (London, UK) on behalf of a client in the pharmaceutical industry. Personal details of respondents were not shared with the authors or the commissioning pharmaceutical company (GW Pharmaceuticals, part of Jazz Pharmaceuticals, Cambridge UK). To ensure anonymization, confidentiality and data protection, only aggregate data was made available to the authors and pharmaceutical company (GW Pharmaceuticals, part of Jazz Pharmaceuticals, Cambridge UK). Authorization for publication of these results was requested and obtained by the Local ethical committee of Regione Calabria (Auth. n. 33/2023).

### Recruitment

HCPs involved in the survey were part of a panel held by the healthcare market research firm GRG Health (New Delhi, India) (Fig. S1). These HCPs had previously opted in to receive requests to participate in surveys and provided their written informed consent to participate in this survey (double consent). Respondents who did not provide their consent were not able to proceed with completing the questionnaire. A quality check was performed to confirm the details of the respondents, and validated participants were invited to take part in the survey via email. A pre-defined quota of HCPs was decided upon prior to starting recruitment, with a plan to recruit a pre-defined number of HCPs from each medical speciality and country (Table 1). Once this quota was met for each medical speciality in each country, no further HCPs were approached. The pre-defined quota contained a greater number of neurologists than HCPs from other medical specialities because the two most recently approved cannabis-based medicines have licensed indications for the treatment of neurological disorders (European Monitoring Centre for Drugs and Drug Addiction 2018). The countries from which HCPs were surveyed were selected based on initial interactions between the commissioning pharmaceutical company (GW Pharmaceuticals, part of Jazz Pharmaceuticals, Cambridge UK) and representatives from the participating countries.

**Table 1:** Number of HCPs from each country of practice and medical speciality who completed the survey

		**Medical speciality**					
		**Neurologist**	**Psychiatrist**	**General practitioner**	**Pharmacist**	**Nurse**	**Total**
**Country of practice**	**Australia**	50	0	0	0	0	50
	**Austria**	30	0	30	30	30	120
	**Brazil**	50	0	0	0	0	50
	**Denmark**	30	0	30	30	30	120
	**France**	30	30	30	30	30	150
	**Germany**	30	30	30	30	30	150
	**Israel**	50	0	0	0	0	50
	**Italy**	30	30	30	30	30	150
	**Japan**	50	0	0	0	0	50
	**Mexico**	50	0	0	0	0	50
	**South Korea**	50	0	0	0	0	50
	**Spain**	30	30	30	30	30	150
	**Sweden**	30	0	30	30	30	120
	**Switzerland**	30	0	30	30	30	120
	**Taiwan**	50	0	0	0	0	50
	**United Kingdom**	30	30	30	30	30	150
	**Total**	620	150	270	270	270	1580

*HCP* healthcare professional

### Eligibility for survey participation

Before commencing the survey, respondents were informed that they could exit at any time, that their identity would be kept confidential, and that their responses would be anonymized. They were also asked to agree to keep information contained within the survey confidential. Respondents were then asked screening questions to determine their eligibility to proceed with the survey. To be eligible to participate further, HCPs had to be currently practising as a neurologist, psychiatrist, general practitioner, pharmacist, or nurse; have practised in their current medical speciality for 3–35 years (inclusive; rationale for inclusion was to ensure respondents had adequate experience of interacting with patients, and to increase the likelihood that they would be in regular contact with patients); be spending ≥ 70% of time in direct patient care (excluding pharmacists). Respondents were also required to agree not to use external resources to help answer any of the questions, confirm that they were not employed directly by a pharmaceutical company and have not participated in cannabis-related market research within the past month.

### Data collection and analyses

The survey took approximately 30 min to complete. All data were collected between November 2020 and February 2021. During a soft launch period, the live survey was completed by a subset of respondents and thoroughly checked before the survey was made available to further respondents. Upon completion of the survey, respondents were paid an honorarium (about 50GBP or equivalent) in line with fair market value guidelines appropriate for each medical speciality and country, which were reviewed and approved by the commissioning pharmaceutical company (GW Pharmaceuticals, part of Jazz Pharmaceuticals, Cambridge UK). For most questions, respondents were asked to rate their opinion on a scale of 1–5, 1–7, or 1–10. Higher scores implied a higher level of interest, knowledge or confidence, or agreement with a statement, depending on the question asked. Mean values are presented alongside standard deviation (SD); median values are presented alongside the interquartile range (IQR). No statistical analysis plan was defined during the design of the survey and therefore analyses throughout are post hoc. Post hoc *t*-tests were used to test for nominally significant statistical differences between groups. Comparisons were drawn between HCPs from each medical speciality (for example, neurologists were individually compared with psychiatrists, general practitioners, pharmacists and nurses), between neurologists situated inside and outside of Europe, and between HCPs who had or had not prescribed, dispensed or recommended any regulatory approved cannabis-based medicines within the past year.* P* < 0.01 was used as the significance threshold.

### Survey questions and data

The data reported in the main body of this publication are from survey questions relating to the attitudes, confidence, and knowledge of HCPs on the use of cannabis-based products in healthcare. The survey questions reported on in this manuscript can be found in Additional file 1. The raw survey data used to draw the conclusions in this manuscript can be found in.

Additional file 2.

## Results

### Respondent characteristics

In total, 5872 HCPs were contacted about their interest in completing this survey until recruitment was completed (Table S1). Of the HCPs contacted, 28.5% (n = 1671) agreed to take part via email and were then screened for their eligibility to participate. 5.4% (n = 91) of these HCPs were not eligible to take part or chose not to complete the survey (Fig. S1). 1580 HCPs from 16 countries (comprising Australia, Austria, Brazil, Denmark, France, Germany, Israel, Italy, Japan, Mexico, South Korea, Spain, Sweden, Switzerland, Taiwan, and the United Kingdom) and from five different specialities (neurologists, psychiatrists, general practitioners, pharmacists, and nurses; Table 1) completed all required questions in the survey. Neurologists were the only medical speciality surveyed from countries outside Europe (Table 1).

At the time of completing the survey, respondents had spent a mean of 11.1 years (SD = 7.0) practising in their medical speciality. The mean proportion of time HCPs spent in direct patient care was 85.3% (SD = 8.2), and they reported personally dealing with a mean of 128.6 patients (SD = 63.2) over the course of a month. The neurologists and psychiatrists surveyed were also asked about the proportion of time spent seeing patients in different practice settings. Most of their time was spent practising in university/teaching hospitals (Table S2).

### Attitudes of HCPs about *cannabis*-based products in healthcare

In response to being asked how interesting they felt cannabis-based products were in healthcare in general, the median score among all respondents was 9 (IQR, 8–10) out of 10, representing a high level of interest (Table 2). Respondents were also asked to what extent they agree that cannabis-based products hold therapeutic potential for some patients, and the median score was 6 (IQR, 5–6) out of 7 (Table 2).

**Table 2:** Attitudes of HCPs about cannabis-based products in healthcare

Question that survey participants (N = 1580) were asked	Scale		Median score (IQR)
How interesting do you feel cannabis-based products are in healthcare?	0–10 (Not at all interesting–extremely interesting)		9 (8–10)
Thinking about cannabis-related products, how do you feel with regard to the following statement?	Cannabis-based products hold therapeutic potential for some patients that I think is important	1–7 (Strongly disagree–strongly agree)	6 (5–6)
	I am knowledgeable about the range of cannabis-based products in healthcare		6 (5–6)
	Cannabis-based products in healthcare are all much the same		3 (2–4)
To what extent do you agree that the way cannabis-based products act in the human body is well understood?	1–5 (Completely disagree–completely agree)		4 (4–4)

*HCP* healthcare professional, *IQR* interquartile range

When asked to what extent they agree that they are knowledgeable about the range of cannabis-based products in healthcare, the median score was 6 (IQR, 5–6) out of 7 (Table 2). HCPs were also asked to what extent they agree that the way cannabis-based products act in the body is understood, to which a score of 4 (IQR, 4–4) out of 5 was recorded (Table 2). When asked to what extent they agree that cannabis-based products in healthcare are all much the same, the median score was 3 (IQR, 2–4) out of 7 (Table 2).

### Interaction of HCPs with patients about *cannabis*-based products and desire for more information

**Fig. 1:**
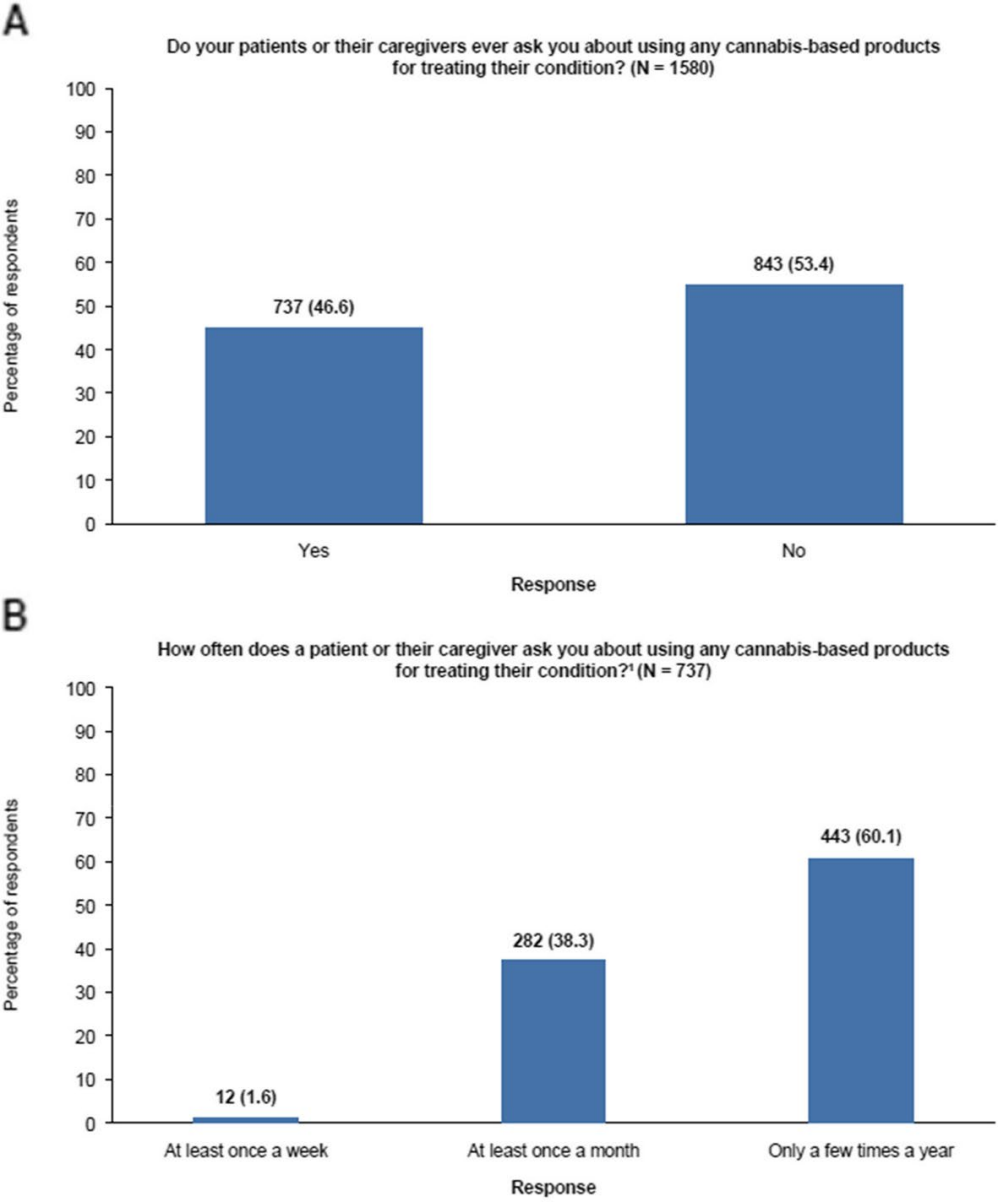
Frequency of HCPs’ interactions with patients about cannabis-based products. ^1^Only the 737 respondents who answered ‘Yes’ to the question “Do your patients or their caregivers ever ask you about using any cannabis-based products for treating their condition?” were asked this question. HCP: healthcare professional

Just under half (46.6%; n = 737) of HCPs surveyed stated that their patients or their patients’ caregivers ask about using cannabis-based products to treat their condition (Table 3). However, when asked to state the frequency of such questions, 60.1% (n = 443) of HCPs surveyed indicated that they were only asked a few times a year, while 38.3% (n = 282) stated they were asked at least once a month and only 1.6% (n = 12) said they were asked at least once a week (Fig. 1).

**Table 3:** Confidence and educational needs of HCPs when interacting with patients about cannabis-based products

Question that survey participants (N = 1310) were asked		Scale	Median score (IQR)
How comfortable are you in discussing cannabis-based products with your patients?		1–7 (Not at all comfortable–extremely comfortable)	6 (6–6)
How confident are you in providing your patients with the following information, so that you can together make a well-informed shared decision?	The legality and regulation of different cannabis-based products in your country	1–7 (Not at all confident–extremely confident)	6 (6–6)
	The evidence underpinning the risks and benefits of different cannabis-based products		5 (3–6)
To what extent do you agree with the following statement? I would feel more confident prescribing (dispensing) or recommending a cannabis-based product if it was reviewed and approved by a medicines regulator (e.g. FDA, EMA), compared with a cannabis-based product that does not have marketing authorization/approval^a^		1–7 (Not at all–completely agree)	6 (6–6)
To what extent do you feel that you would benefit from more information with regards to the following types of information?	Information on the legality and regulation of different cannabis-based products	1–7 (No benefit at all–extremely beneficial)	6 (6–6)
	Information on the neurobiology of cannabis-based products		6 (5–6)
	Information on the scientific evidence of risks and benefits of different cannabis-based products		6 (5–6)
	Information on the different types of product available, their cannabinoid content, strength and pharmacokinetics, and drug interaction potential		6 (5–6)

*EMA* European Medicines Agency, *FDA* US Food and Drug Administration, *HCP* healthcare professional, *IQR* interquartile range

^a^Of the survey questions in this table, pharmacists were only asked the question marked with an asterisk. For this question, N = 1580. Pharmacists were asked if they would feel more confident dispensing (rather than prescribing, as all other medical specialities were)

When asked how comfortable they are in discussing cannabis-based products with their patients, the median score across all respondents was 6 (IQR, 6–6) out of 7, suggesting a high level of comfort (Table 3). Similarly, when asked how confident they are in providing their patients with information on the legality and regulations of different cannabis-based products in their country of practice, the HCPs returned a median score of 6 (IQR, 6–6) out of 7 (Table 3). A median score of 5 (IQR, 3–6) out of 7 was obtained when the HCPs were asked about their confidence in providing their patients with information on the evidence underpinning the risks and benefits of different cannabis-based products (Table 3). HCPs strongly agreed that they would feel more confident prescribing cannabis-based products if they had been reviewed and approved by medicines regulators, returning a median score of 6 (IQR, 6–6) out of 7 (Table 3).

HCPs were also asked to what extent they would benefit from receiving further information on various topics relevant to cannabis-based products. For all the topics asked in this question, the HCPs surveyed returned a median score of 6 out of 7 (IQR values for each question can be found in Table 3).

### Opinions of HCPs on factors that contribute to patient safety in the context of *cannabis*-based products

**Fig. 2:**
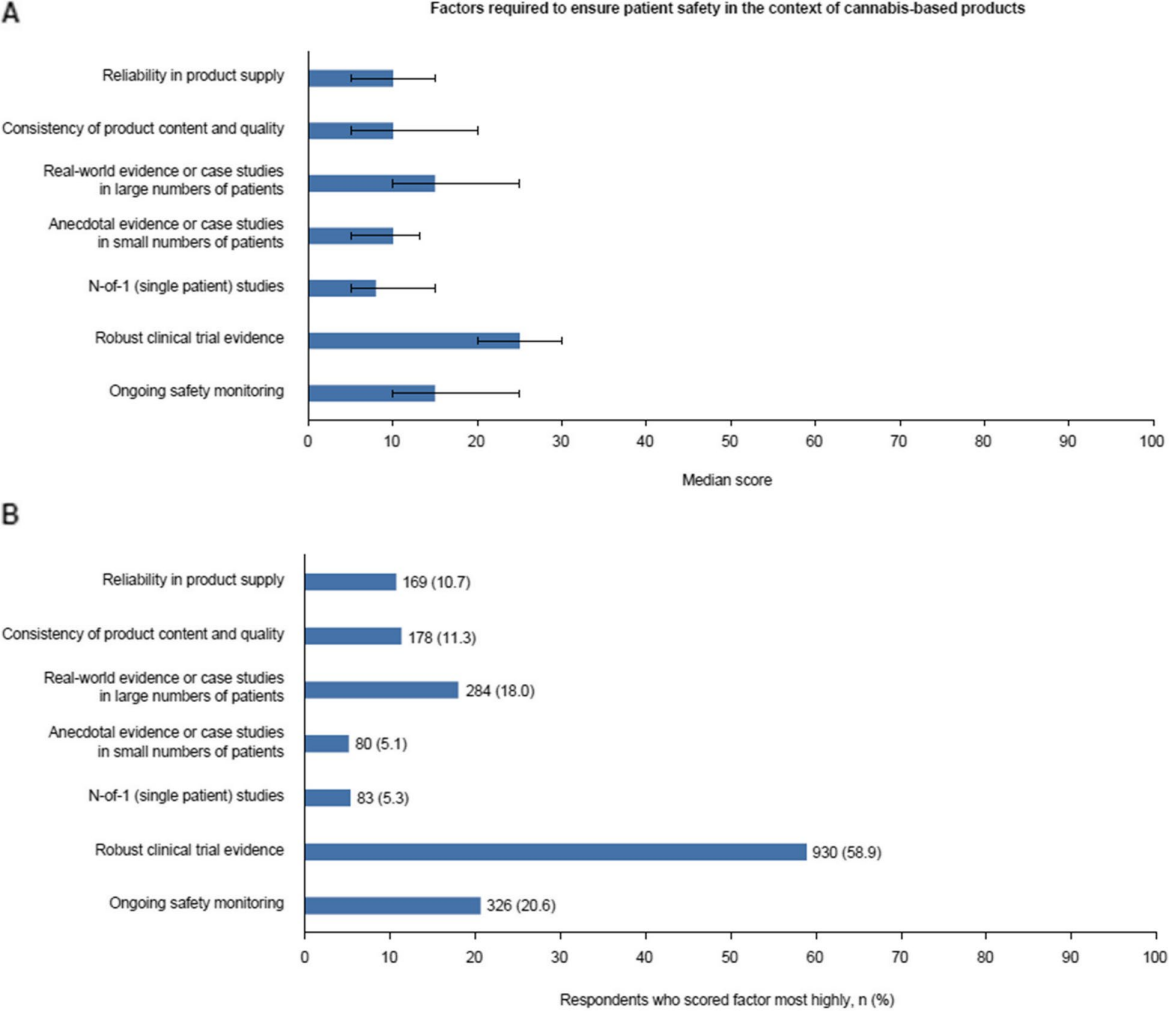
Factors required to ensure patient safety in the context of cannabis-based products. Respondents were asked to score the extent to which various factors should be required to ensure patient safety in relation to cannabis-based products (higher scores indicate higher importance). Respondents scored each factor 0–100, with scores being required to sum 100. Presented is **A**) the median score for each factor and **B**) the number of respondents who scored each factor most highly. Error bars represent the IQR. IQR: interquartile range

When considering how appropriate it is to prescribe, dispense, or recommend a cannabis-based product to their patients, HCPs must consider the types of evidence that exist to support their efficacy and safety profiles. Respondents were asked to score the relative importance of various factors that contribute to patient safety by allocating a proportion of a total of 100 points to the different areas based on the extent of their importance. The HCPs surveyed scored robust clinical trial evidence most highly, with a median score of 25 (IQR, 20–30) and with 58.9% (n = 930) of respondents selecting it as their highest scoring factor (Fig. 2A and B). Ongoing safety monitoring and real-world evidence or registries in large numbers of patients were assigned the joint second highest score, both with medians of 15 (IQR, 10–25) (Fig. 2A). Of the options provided, N-of-1 (single patient) studies scored the lowest, with a median of 8 (IQR, 5–15; Fig. 2A).

### Comparisons between medical specialities

Additional post hoc analyses were conducted between HCP groups and the overall sample. Responses from the groups were similar, with few nominally significant differences observed between HCPs from the different medical specialities (Tables S3–6). Summarizing, neurologists are significantly more likely to consider cannabis-based products as more interesting that GPs, pharmacists and nurses. Neurologists consider themselves more knowledgeable about the range of these products than GP’s, pharmacists and nurses (Table S3). Neurologists are significantly more comfortable than psychiatrists in discussing these products and significantly more confident about legality and regulation than psychiatrists (Table S5). On the other hand, psychiatrists significantly believe that robust clinical trial evidence is needed in comparison to neurologists (Table S6). Notably, psychiatrists reported being asked most frequently by their patients or their patients’ caregivers about the use of cannabis-based products for treating their condition. 58.6% (n = 41/150) of psychiatrists indicated that they were asked at least once a month; although there was no statistical significance in comparison to the overall HCP population surveyed (38.3%; n = 282/737).

### Comparisons between European and non-European neurologists

Neurologists were the only speciality surveyed with representation from outside Europe (Table 1); therefore, additional post hoc analyses were conducted to determine whether there were differences between neurologists practising in European countries and those practising in countries outside Europe. Few differences of nominal significance were observed between neurologists from the two different regions (Tables S7–10).

### Impact of prescribing, dispensing and recommending *cannabis*-based products on HCP confidence and interest in receiving further information

During the survey, HCPs were asked if they had prescribed, dispensed, or recommended any regulatory approved cannabis-based medicines within the past year. In total, 36.3% (n = 573) of the HCPs stated they had, while 63.7% (n = 1007) stated they had not. To explore whether this influenced the respondents’ confidence in discussing cannabis-based products with patients and their interest in receiving further information, a comparison was made between answers from respondents who stated that they had prescribed, dispensed, or recommended a regulatory approved cannabis-based medicine within the past year and respondents who had not. Overall, there were very few nominally significant differences between the two groups. However, when asked about how confident they felt when providing patients with information on the evidence underpinning the risks and benefits of different cannabis-based products, HCPs who had prescribed, dispensed, or recommended cannabis tended to rate their confidence as lower than those who had not, with a median score of 3 (IQR, 3–5) out of 7, compared with 5 (IQR, 4–6) out of 7, respectively (*P* < 0.01).

## Discussion

To the authors’ knowledge, this is the largest survey evaluating the attitudes held by HCPs about cannabis-based products in healthcare, at the time of writing. The results suggest that HCPs are highly interested in, and optimistic about, the therapeutic potential of cannabis-based products. HCPs considered robust clinical trial data to be the most important factor for ensuring patient safety in relation to cannabis-based products. While HCPs felt knowledgeable and confident when discussing cannabis-based products with their patients or their patients’ caregivers, they also indicated that they would benefit from receiving more information on a range of topics in relation to these products. Survey responses were generally consistent across HCPs from different medical specialities.

One result the survey highlighted is the importance of robust clinical trial evidence to HCPs with respect to patient safety, with 58.9% (n = 930) respondents considering it as the most important contributing factor in the context of cannabis-based products. This complements the observation that HCPs feel they would benefit from receiving more information on a variety of topics around cannabis-based products, irrespective of their recent experience of prescribing cannabis-based medicines. These findings advocate the design of high-quality clinical trial programs, which are necessary to reliably evaluate the efficacy and safety of cannabis-based products for use in clinical practice, just as they are for all products irrespective of their origin. These types of data are required for approval by medicines regulators, which the surveyed HCPs indicated was an important contributor to their confidence when prescribing, dispensing, or recommending cannabis-based medicines. Dissemination of the latest data and reliable information about cannabis-based products and medicines could be achieved through the provision of up-to-date educational materials and programs that are tailored to HCPs (Szaflarski et al. 2020).

The finding that HCPs expressed a high level of confidence when discussing cannabis-based products with their patients contrasts the low-tomoderate levels of confidence indicated by HCPs in previous research (Hordowicz et al. 2021; Gardiner et al. 2019; St Pierre et al. 2020; Kruger et al. 2021). This may represent varying confidence levels of HCPs from different medical specialities and countries, especially in studies where those surveyed were from a single medical speciality or geographical region, such as the US (Philpot et al. 2019; Elliott et al. 2020; Arnfinsen and Kisa 2020; Jacobs et al. 2019; Chan 2017). Alternatively, this may be due to differences in survey design, which has previously been acknowledged as a challenge when drawing cross-study comparisons of the attitudes of HCPs towards cannabis-based products (Gardiner et al. 2019). One notable observation is that respondents returned a score reflecting a high level of perceived confidence when discussing the evidence underpinning the risks and benefits of different cannabis-based products with patients. Given there are many questions that remain unanswered about cannabinoids and how they interact with the endocannabinoid system and other body systems (Cristino et al. 2020), this may represent an over-confidence among the respondents. This would be consistent with the previous finding that HCPs reported significant knowledge gaps about cannabis-based products in healthcare (Szaflarski et al. 2020). These results suggest that recent experience of prescribing, dispensing, or recommending regulatory approved cannabis-based medicines is not an indicator of confidence about the use of cannabis-based products in healthcare. This is somewhat surprising but may represent an appreciation from HCPs who work regularly with cannabis-based products and medicines that much remains to be understood about products and medicines derived from cannabis.

Almost half of the HCPs surveyed reported that they received questions from patients about using cannabis-based products to treat their condition. Interestingly, of those medical specialities surveyed, psychiatrists were asked most frequently about cannabis-based products. This may suggest that patients with psychiatric conditions are currently more aware of, and interested in, cannabis-based products for the management of their condition than other patient populations. Relevant to this observation, real-world evidence from Italy showed that the prescription frequency of psychiatric medications decreased following the unintended liberalization of a product marketed as containing CBD (referred to as ‘cannabis light’ locally) (Carrieri et al. 2020). This shows the relevance of cannabis-based products to this patient population, where a proportion of patients chose ‘self-medication’ over seeking advice and treatment from HCPs (Carrieri et al. 2020). One area for future research will be to see whether the number of questions from patients increases alongside the increasing number and availability of cannabis-based products.

A strength of this study is the size and diversity of the HCP population surveyed, which included HCPs practising in multiple medical specialities across 16 countries from Asia, Europe, Oceania, South America, and the Middle East. HCPs from the medical specialities included in this study were surveyed based on the understanding that they were most likely to receive questions from patients about cannabis-based products in a healthcare setting. The diversity of the population surveyed enabled differences to be investigated in attitudes, knowledge, and confidence that exists between HCPs from different medical backgrounds and from different regions. Perhaps surprisingly, post hoc testing identified few differences between HCPs from the different medical specialities. As neurologists and psychiatrists work most closely with most of the medical conditions where cannabis-based medicines have been approved for use by medicines regulators (European Monitoring Centre for Drugs and Drug Addiction 2018), it may have been expected that they would score some of the questions differently to the other respondents,however, this was not the case. Furthermore, the similarity in responses between neurologists practising inside and outside Europe suggests that, despite regulations around cannabis-based products such as medical cannabis differing considerably between countries, the views of HCPs from different regions appear generally consistent. The results of this study suggest that HCPs from all the medical specialities surveyed here would benefit from receiving more evidence-based information around cannabis-based products in healthcare. Overall, the results of our study are also in line with the recently published results by Bawa et al. (2022) in a cross-sectional study including 505 Australian General practitioners on-line surveyed by a 42-item questionnaire adapted from a previous survey studied in 2017 (Karanges et al. 2018). Also, the most recent study by this group underlines the need for improved training and education.

There are also limitations of this study. The statistical analysis conducted was not pre-defined, and therefore analysis of the study data was limited to post hoc analyses only. Future hypothesis-driven studies are required to build on the findings reported in this study.

Although a diverse population of HCPs was surveyed, a pre-defined quota of HCPs from different countries and medical specialities was set prior to the onset of this study. As such, the number of HCPs from these countries and medical specialities will not be proportionally representative of all HCPs globally. The survey did not include respondents from the USA or Canada. Medical cannabis programs have been in place for some time in these countries, and it would be of interest to understand the influence this had on the attitudes and behaviours held by HCPs. Information about respondents, including gender, age, ethnicity, and the therapy area in which each HCP specialized at the time of completing the survey was not recorded, meaning that responses could not be broken down based on these characteristics; future hypothesis-driven research could investigate attitudes of HCPs based on characteristics such as these. HCPs were recruited from a panel who had voluntarily signed up to participate in surveys such as this, potentially selecting for HCPs with particular attitudes or excluding those from institutions that do not allow employees to participate in surveys in which they are paid an honorarium. Overall, the above issues constitute potential sample bias which limit the validity of the results obtained including a low external validity. Nevertheless, the present results still represent at least a relevant quote of HCPs and related information.

It should be noted that he purpose of this market research was framed to potential respondents using the following introductory wording: “The purpose of this research is to gain insights into the awareness, perception and use of cannabinoids for medical use among healthcare professionals.” Therefore, while there may be a selection bias towards HCPs who were more interested in the topic of cannabinoids for medical use, there was no pressure towards, or positive or negative opinion, within the questionnaire framing. No mention of the commissioning pharmaceutical company (GW Pharmaceuticals, part of Jazz Pharmaceuticals, Cambridge, UK) was made at any point during the invitation or questionnaire but was presented as being conducted by Cello Health Insight, an independent marketing research company specialising in the pharmaceutical and healthcare industry.

The fast-moving nature of cannabinoid science may also mean that the attitudes of HCPs will quickly change, advocating further research to understand the changing attitudes of HCPs towards cannabis-based products over time. Finally, as with any survey, caution must be exercised with the interpretation of the results. Although there was generally a high level of consistency between the responses to questions, individuals may interpret questions in different ways.

## Conclusions

These findings suggest that HCPs are interested in and optimistic about the use of cannabis-based products in healthcare. Further education and high-quality evidence around cannabis-based products can help to ensure HCPs remain well-informed about this rapidly advancing area of medical science. Furthermore, access to educational resources on cannabis-based products may increase their confidence in discussing the benefits and risks of these with their patients and their patients’ caregivers.

### Supplementary Information

Supplementary Material 1.

## Data Availability

The dataset supporting the conclusions of this article is included within the article and its additional files.

## Abbreviations

AIDS: Acquired immune deficiency syndrome
BHBIA: British Healthcare Business Intelligence Association
CBD: Cannabidiol
CBG: Cannabigerol
CBDV: Cannabidivarin
DS: Dravet syndrome
EMA: European Medicines Agency
EphMRA: European Pharmaceutical Market Research Association
FDA: United States Food and Drug Administration
HCP: Healthcare professional
IQR: Interquartile range
LGS: Lennox–Gastaut syndrome
SD: Standard deviation
THC: Delta-9-tetrahydrocannabinol
TSC: Tuberous sclerosis complex
