# Supplementary material for: The attitudes, knowledge and confidence of healthcare professionals about cannabis-based products
Source: J Cannabis Res. 2024 Jul 24;6:32. doi: 10.1186/s42238-024-00242-y (PMC11267914; doi:10.1186/s42238-024-00242-y)
Supplement: Supplementary file 1 — Supplementary Material 1. [file 42238_2024_242_MOESM1_ESM.docx]

**Supplementary materials**

**The attitudes, knowledge and confidence of healthcare professionals about
cannabis-based products**

Emilio Russo, Paula Martinez Agredano, Peter Flachenecker, Charlotte Lawthom, Duncan Munro, Chandni Hindocha, Makarand Bagul, Eugen Trinka

| **Supplementary figure/table** | **Page(s)** |
| --- | --- |
| **Figure S1** The process used to conduct the survey described in this study, from recruitment to completion | 1 |
| **Table S1** Overview of HCPs who were contacted about completing the survey | 2–3 |
| **Table S2** Proportion of time spent by neurologists and psychiatrists in different practice settings | 4 |
| **Tables S3–6** Survey data broken down by medical speciality of HCP | 5–13 |
| **Tables S7–10** Survey data broken down by neurologists inside and outside of Europe | 14–21 |
| **Table S11** Attitudes and confidence of HCPs about non-regulatory approved cannabis-based products and regulatory approved cannabis-based medicines in healthcare | 22–23 |
| **Table S12** Patient questions about cannabis-based products | 24 |
| **Table S13** Knowledge of HCPs regarding cannabinoids and the cannabis plant | 25 |
| **Table S14** Confidence of respondents in the body of evidence for the safety and efficacy of cannabis-based products in healthcare based on their regulatory approval status | 26 |
| **Table S15** Confidence of respondents on the legality of cannabis-based products in healthcare | 27 |
| **Table S16** Attitudes of HCPs towards the prescription or access of non-regulatory approved cannabis-based products | 28 |
| **Table S17** Preferred method of receiving information on the legality, regulations, neurobiology, efficacy and safety, and clinical pharmacology of cannabis-based products | 29–30 |

**
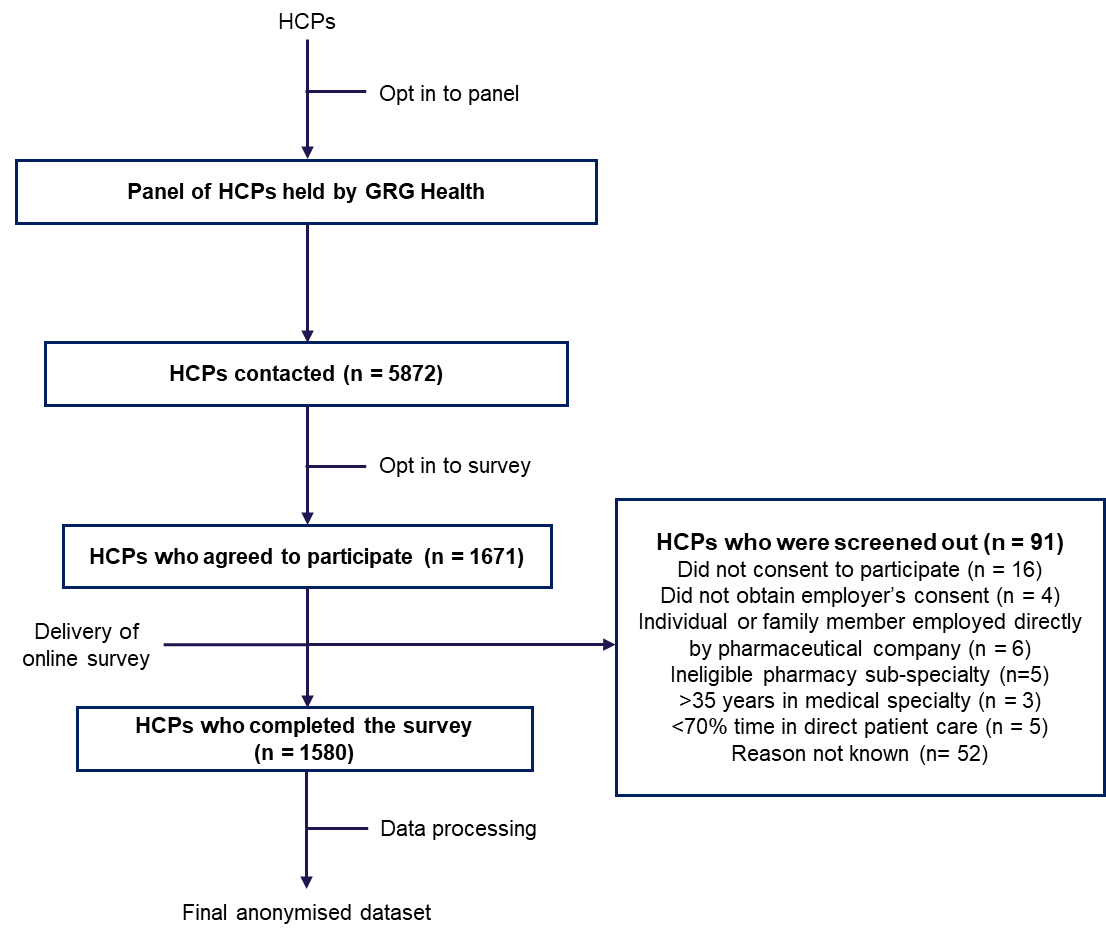
**

**Fig S1** The process used to conduct the survey described in this study, from recruitment to completion.
HCP: healthcare professional

**Table S1** Overview of HCPs who were contacted about completing the survey

|  | | **Medical speciality** | | | | | |
| --- | --- | --- | --- | --- | --- | --- | --- |
|  |  | **Neurologists** | **Psychiatrists** | **General practitioners** | **Pharmacists** | **Nurses** | **Total** |
| **Country**  **of practice** | **Australia** | 220 | 0 | 0 | 0 | 0 | **220** |
|  | **Austria** | 110 | 0 | 106 | 99 | 113 | **428** |
|  | **Brazil** | 270 | 0 | 0 | 0 | 0 | **270** |
|  | **Denmark** | 95 | 0 | 90 | 85 | 90 | **360** |
|  | **France** | 90 | 96 | 74 | 105 | 81 | **446** |
|  | **Germany** | 90 | 98 | 96 | 120 | 102 | **506** |
|  | **Israel** | 218 | 0 | 0 | 0 | 0 | **218** |
|  | **Italy** | 85 | 86 | 80 | 100 | 84 | **435** |
|  | **Japan** | 310 | 0 | 0 | 0 | 0 | **310** |
|  | **Mexico** | 240 | 0 | 0 | 0 | 0 | **240** |
|  | **South Korea** | 260 | 0 | 0 | 0 | 0 | **260** |
|  | **Spain** | 86 | 85 | 84 | 110 | 85 | **450** |
|  | **Sweden** | 113 | 0 | 115 | 140 | 122 | **490** |
|  | **Switzerland** | 120 | 0 | 125 | 120 | 115 | **480** |
|  | **Taiwan** | 280 | 0 | 0 | 0 | 0 | **280** |
|  | **United Kingdom** | 106 | 80 | 89 | 80 | 124 | **479** |
|  | **Total** | **2693** | **445** | **859** | **959** | **916** | **5872** |

HCP: healthcare professional

**Table S2** Proportion of time spent by neurologists and psychiatrists in different practice settings

|  | **Mean proportion of time spent in practice setting, % (standard deviation)** | |
| --- | --- | --- |
| **Practice setting** | **Neurologists (n = 620)** | **Psychiatrists (n = 150)** |
| **University/teaching hospital** | 54.2 (49.6) | 36.6 (48.1) |
| **Community hospital** | 20.7 (40.4) | 21.5 (41.1) |
| **Private hospital** | 12.1 (32.5) | 15.8 (36.4) |
| **Office-based clinic** | 1.4 (11.4) | 5.3 (22.3) |
| **Community practice** | 2.1 (14.3) | 3.0 (16.1) |
| **Private practice** | 9.2 (28.7) | 17.8 (37.9) |
| **Other** | 0.3 (5.68) | 0.0 (0.0) |

Only neurologists and psychiatrists were asked this question

**Table S3** Attitudes of HCPs about cannabis-based products in healthcare (by medical speciality of HCP)

| **Question that survey participants were asked** | | **Scale** | **Median score (IQR)** | | | | |
| --- | --- | --- | --- | --- | --- | --- | --- |
|  |  |  | **Neurologists**  **(n = 620)** | **Psychiatrists (n = 150)** | **General practitioners (n = 270)** | **Pharmacists (n = 270)** | **Nurses (n = 270)** |
| How interesting do you feel cannabis-based products are in healthcare? | | 0–10  (Not at all interesting–extremely interesting) | 10 (9–10)^cde^ | 9 (9–10)^e^ | 9 (8–10) | 8 (8–10) | 8 (8–10) |
| Thinking about cannabis-related products, how do you feel with regard to the following statement? | Cannabis-based products hold therapeutic potential for some patients that I think is important | 1–7 (Strongly disagree– strongly agree) | 6 (5–6) | 6 (5–6)^e^ | 6 (5–6)^e^ | 6 (5–6) | 6 (5–6) |
|  | I am knowledgeable about the range of cannabis‑based  products in healthcare |  | 6 (5–6)^cde^ | 6 (5–6)^de^ | 6 (5–6)^e^ | 6 (5–6) | 6 (5–6) |
|  | Cannabis-based products in healthcare are all much the same |  | 3 (2–5) | 4 (3–5)^cd^ | 3 (2–4) | 3 (2–4) | 3 (2–4) |
| To what extent do you agree that the way  cannabis-based products act in the human body  is well understood? | | 1–5  (Completely disagree–completely agree | 4 (4–4) | 4 (4–4) | 4 (4–4) | 4 (4–4) | 4 (4–4) |

Letters in superscript represent nominally significant differences between groups (a: neurologists, b: psychiatrists, c: general practitioners, d: pharmacists, e: nurses). Post hoc statistical testing was performed using *t*-tests. *P* < 0.01 was used as the significance threshold. All *P* values calculated were nominal.
HCP: healthcare professional, IQR: interquartile range

**Table S4** Frequency of HCPs’ interactions with patients about cannabis-based products (by medical speciality of HCP)

| **Question that survey participants were asked** | **Response** | **Proportion of HCPs, n (%)** | | | | |
| --- | --- | --- | --- | --- | --- | --- |
|  |  | **Neurologists**  **(n = 620)** | **Psychiatrists (n = 150)** | **General practitioners (n = 270)** | **Pharmacists (n = 270)** | **Nurses (n = 270)** |
| Do your patients or their caregivers ever ask you about using any cannabis-based products for treating their condition? | Yes | 279 (45.0) | 70 (46.7) | 136 (50.4) | 136 (50.4) | 116 (43.0) |
|  | No | 341 (55.0) | 80 (53.3) | 134 (49.6) | 134 (49.6) | 154 (57.0) |
| How often does a patient or their caregiver ask you about using any cannabis-based products for treating their condition?^a^ | At least once a week | 4 (1.4) | 1 (1.4) | 0 (0.0) | 5 (3.7) | 2 (1.7) |
|  | At least once a month | 101 (36.2) | 41 (58.6) | 47 (34.6) | 46 (33.8) | 47 (40.5) |
|  | Only a few times a year | 174 (62.4) | 28 (40.0) | 89 (65.4) | 85 (62.5) | 67 (57.8) |

^a^Only the 279 neurologists, 70 psychiatrists, 136 general practitioners, 136 pharmacists and 116 nurses who answered ‘Yes’ to the question “Do your patients or their caregivers ever ask you about using any cannabis-based products for treating their condition?” were asked this question.

Post hoc statistical testing was performed using *t*-tests. *P* < 0.01 was used as the significance threshold. All *P* values calculated were nominal
HCP: healthcare professional

**Table S5** Confidence of HCPs when interacting with patients about cannabis-based products, and the extent to which they feel that they would benefit from receiving more information on these (by medical speciality of HCP)

| **Question that survey participants were asked** | | **Scale** | **Median score (IQR)** | | | | |
| --- | --- | --- | --- | --- | --- | --- | --- |
|  |  |  | **Neurologists**  **(n = 620)** | **Psychiatrists (n = 150)** | **General practitioners (n = 270)** | **Pharmacists (n = 270)** | **Nurses (n = 270)** |
| How comfortable are you in discussing  cannabis-based products with your patients? | | 1–7  (Not at all comfortable –extremely comfortable) | 6 (6–6)^be^ | 6 (5–6) | 6 (6–6)^e^ | n/a^f^ | 6 (5–6) |
| How confident are you in providing your patients with the following information,  so that you can together  make a well-informed  shared decision? | The legality and regulation of different cannabis‑based products in your country | 1–7 (Not at all confident–extremely confident) | 6 (6–6)^be^ | 6 (5–6) | 6 (6–6)^be^ | n/a^f^ | 6 (5–6) |
|  | The evidence underpinning the risks and benefits of different cannabis-based products |  | 4 (3–6) | 5 (3–6) | 6 (3–6) | n/a^f^ | 4 (3–5) |
| To what extent do you agree with the following statement?  I would feel more confident prescribing (dispensing) or recommending a cannabis-based product if it was reviewed and approved by a medicines regulator (e.g. FDA, EMA), compared with a cannabis-based product that does not have marketing authorisation/approval | | 1–7 (Not at all–completely agree) | 6 (6–6) | 6 (5–6) | 6 (6–6) | 6 (6–6) | 6 (5–6) |
| To what extent do you feel that you would benefit from more information with regards to the following types  of information? | Information on the legality and regulation of different  cannabis-based products | 1–7 (No benefit at all–extremely beneficial) | 6 (6–6)^e^ | 6 (6–6) | 6 (6–6)^e^ | n/a^f^ | 6 (5–6) |
|  | Information on the neurobiology of  cannabis-based products |  | 6 (5–6)^e^ | 6 (5–6) | 6 (5–6)^e^ | n/a^f^ | 6 (5–6) |
|  | Information on the scientific evidence and risk of different cannabis-based products |  | 6 (5–6)^e^ | 6 (5–6) | 6 (5–6) | n/a^f^ | 6 (5–6) |
|  | Information on the different types of product available, their cannabinoid content, strength and pharmacokinetics, and drug interaction potential |  | 6 (5–6)^e^ | 6 (5–6) | 6 (5–6)^e^ | n/a^f^ | 6 (5–6) |

^f^Pharmacists were not asked these questions.
Letters in superscript represent nominally significant differences between groups (a: neurologists, b: psychiatrists, c: general practitioners, d: pharmacists, e: nurses). Post hoc statistical testing was performed using *t*-tests. *P* < 0.01 was used as the significance threshold. All *P* values calculated were nominal.
EMA: European Medicines Agency, FDA: US Food and Drug Administration, HCP: healthcare professional, IQR: interquartile range

**Table S6** Factors that HCPs consider important to ensure patient safety in the context of cannabis-based products (by medical speciality of HCP)

| **Question that survey participants were asked** | | **Scale** | **Median score (IQR)** | | | | |
| --- | --- | --- | --- | --- | --- | --- | --- |
|  |  |  | **Neurologists**  **(n = 620)** | **Psychiatrists (n = 150)** | **General practitioners (n = 270)** | **Pharmacists (n = 270)** | **Nurses (n = 270)** |
| To ensure patient safety,  to what extent should each of the following be required for cannabis-based products? | Ongoing safety monitoring | 0–100 | 15 (10–25) | 15 (10–20) | 15 (10–25) | 15 (10–20) | 15 (10–25) |
|  | Robust clinical trial evidence |  | 25 (20–30)^b^ | 30 (20–30)^e^ | 25 (25–30) | 25 (20–30) | 25 (20–30) |
|  | N-of-1 (single patient) studies |  | 8 (5–15) | 10 (5–15) | 6 (5–12) | 10 (5–15) | 9 (5–15) |
|  | Anecdotal evidence or case studies in small numbers  of patients |  | 10 (5–15) | 10 (5–13.5) | 5 (5–12) | 10 (5–15)^c^ | 10 (5–12) |
|  | Real-world evidence or registries in large numbers of patients |  | 15 (10–25) | 15 (10–20)^acd^ | 20 (12–25)^e^ | 15 (10–25)^b^ | 15 (10–25) |
|  | Consistency of product content and quality |  | 10 (5–20) | 11 (5–20) | 10 (5–20) | 10 (5–15)^abe^ | 10 (5–20) |
|  | Reliability in product supply |  | 10 (5–15) | 10 (5–15) | 10 (5–15) | 10 (5–15) | 10 (5–20) |

Respondents were asked to score the extent to which various factors should be required in order to ensure patient safety in relation to cannabis-based products (higher scores indicate higher importance). Respondents scored each factor 0–100, with scores being required to sum 100.
Letters in superscript represent nominally significant differences between groups (a: neurologists, b: psychiatrists, c: general practitioners, d: pharmacists, e: nurses). Post hoc statistical testing was performed using *t*-tests. *P* < 0.01 was used as the significance threshold. All *P* values calculated were nominal.
HCP: healthcare professional, IQR: interquartile range

**Table S7** Neurologists from within and outside Europe: attitudes about cannabis-based products in healthcare

| **Question that survey participants were asked** | | **Scale** | **Median score (IQR)** | |
| --- | --- | --- | --- | --- |
|  |  |  | **Neurologists from Europe**  **(n = 270)** | **Neurologists from outside Europe**  **(n = 350)** |
| How interesting do you feel cannabis-based products are in healthcare? | | 0–10  (Not at all interesting–extremely interesting) | 10 (9–10) | 10 (9–10) |
| Thinking about cannabis-related products, how do you feel with regard to the following statement? | Cannabis-based products hold therapeutic potential for some patients that I think is important | 1–7 (Strongly disagree– strongly agree) | 6 (5–6) | 6 (5–6) |
|  | I am knowledgeable about the range of cannabis-based products in healthcare |  | 6 (5–6) | 6 (5–6) |
|  | Cannabis-based products in healthcare are all much the same |  | 4 (3–5) | 3 (2–4) |
| To what extent do you agree that the way  cannabis-based products act in the human body  is well understood? | | 1–5  (Completely disagree–completely agree) | 4 (4–4) | 4 (4–4) |

Post hoc statistical testing was performed using *t*-tests. *P* < 0.01 was used as the significance threshold. All *P* values calculated were nominal
HCP: healthcare professional, IQR: interquartile range

**Table S8** Neurologists from within and outside Europe: frequency of interactions with patients about cannabis‑based products

| **Question that survey participants  were asked** | **Response** | **Proportion of HCPs, n (%)** | |
| --- | --- | --- | --- |
|  |  | **Neurologists from Europe**  **(n = 270)** | **Neurologists from outside Europe**  **(n = 350)** |
| Do your patients or their caregivers ever ask you about using any cannabis-based products for treating their condition? | Yes | 126 (46.7) | 153 (43.7) |
|  | No | 144 (53.3) | 197 (56.3) |
| How often does a patient or their caregiver ask you about using any cannabis-based products for treating their condition?^a^ | At least once a week | 1 (0.8) | 3 (2.0) |
|  | At least once a month | 48 (38.1) | 53 (34.6) |
|  | Only a few times a year | 77 (61.1) | 97 (63.4) |

^a^Only the 126 neurologists from Europe and 153 neurologists from outside Europe who answered ‘Yes’ to the question “Do your patients or their caregivers ever ask you about using any cannabis-based products for treating their condition?” were asked this question.
HCP: healthcare professional

**Table S9** Neurologists from within and outside Europe: confidence when interacting with patients about cannabis‑based products, and the extent to which they feel that they would benefit from receiving more information on these

| **Question that survey participants were asked** | | **Scale** | **Median score (IQR)** | |
| --- | --- | --- | --- | --- |
|  |  |  | **Neurologists from Europe**  **(n = 270)** | **Neurologists from outside Europe**  **(n = 350)** |
| How comfortable are you in discussing  cannabis-based products with your patients? | | 1–7  (Not at all comfortable –extremely comfortable) | 6 (6–6) | 6 (6–6) |
| How confident are you in providing your patients with the following information,  so that you can together make a  well-informed  shared decision? | The legality and regulation of different cannabis‑based products in your country | 1–7 (Not at all confident–extremely confident) | 6 (6–6) | 6 (6–6) |
|  | The evidence underpinning the risks and benefits of different cannabis-based products |  | 4 (3–6) | 4 (3–6) |
| To what extent do you agree with the following statement? I would feel more confident prescribing (dispensing) or recommending a cannabis-based product if it was reviewed and approved by a medicines regulator (e.g. FDA, EMA), compared with a cannabis-based product that does not have marketing authorisation/approval | | 1–7 (Not at all–completely agree) | 6 (6–6) | 6 (6–6) |
| To what extent do you feel that you would benefit from more information with regards to the following types of information? | Information on the legality and regulation of different  cannabis-based products | 1–7 (No benefit at all–extremely beneficial) | 6 (5–6) | 6 (5–6) |
|  | Information on the neurobiology of cannabis-based products |  | 6 (5–6) | 6 (5–6) |
|  | Information on the scientific evidence and risk of different cannabis-based products |  | 6 (5–6) | 6 (5–6) |
|  | Information on the different types of product available, their cannabinoid content, strength and pharmacokinetics, and drug interaction potential |  | 6 (5–6) | 6 (5–6) |

Post hoc statistical testing was performed using *t*-tests. *P* < 0.01 was used as the significance threshold. All *P* values calculated were nominal
EMA: European Medicines Agency, FDA: US Food and Drug Administration, HCP: healthcare professional, IQR: interquartile range.

**Table S10** Neurologists from within and outside Europe: factors that are considered important to ensure patient safety in the context of cannabis-based products

| **Question that survey participants were asked** | | **Scale** | **Median score (IQR)** | |
| --- | --- | --- | --- | --- |
|  |  |  | **Neurologists from Europe**  **(n = 270)** | **Neurologists from outside Europe**  **(n = 350)** |
| To ensure patient safety, to what extent should each of the following be required for  cannabis-based products? | Ongoing safety monitoring | 0–100 | 15 (10–20) | 15 (10–25) |
|  | Robust clinical trial evidence |  | 25 (20–30)^a^ | 25 (20–30)^a^ |
|  | N-of-1 (single patient) studies |  | 10 (5–15) | 5 (5–12.75) |
|  | Anecdotal evidence or case studies in small numbers of patients |  | 10 (5–15) | 10 (5–15) |
|  | Real-world evidence or registries in large numbers of patients |  | 15 (10–25) | 15 (10–25) |
|  | Consistency of product content and quality |  | 10 (5–15) | 10 (5–20) |
|  | Reliability in product supply |  | 10 (5–15) | 10 (5–15) |

Respondents were asked to score the extent to which various factors should be required in order to ensure patient safety in relation to cannabis-based products (higher scores indicate higher importance). Respondents scored each factor 0–100, with scores being required to sum 100.
^a^Nominally significant differences between groups. Post hoc statistical testing was performed using *t*-tests. *P* < 0.01 was used as the significance threshold. All *P* values calculated were nominal.
HCP: healthcare professional, IQR: interquartile range

**Table S11** Attitudes and confidence of HCPs about non-regulatory approved cannabis-based products and regulatory approved cannabis-based medicines in healthcare

| **Question that survey participants (N = 1580) were asked** | | **Scale** | **Median (IQR)** |
| --- | --- | --- | --- |
| Thinking about cannabis-related products, how do you feel with regard to the  following statement? | Non-regulatory approved cannabis-based products (‘medical cannabis’) have not yet earned their place in healthcare | 1–7 (Strongly disagree– strongly agree) | 5 (5–6) |
|  | Regulatory approved cannabis-based medicines have not yet earned their place in healthcare |  | 3 (3–5) |
|  | I do not yet fully trust non-regulatory approved cannabis-based products (‘medical cannabis’) in healthcare |  | 5 (3–6) |
|  | I do not yet fully trust regulatory approved  cannabis-based medicines in healthcare |  | 3 (2–3) |
| How confident are you in the scientific evidence base for the use of these categories of products in medicine? | Regulatory approved cannabis-based medicines | 1–7 (Not at all confident–extremely confident) | 6 (6–6) |
|  | Non-regulatory approved cannabis-based products  (‘medical cannabis’) |  | 4 (3–4) |

The below definitions of non-regulatory approved cannabis-based products and regulatory approved cannabis-based medicines were provided and made accessible to respondents while answering these questions:
**Non-regulatory approved cannabis-based products:** cannabis-based products used for medical purposes via prescription but not approved by medicines regulators – often referred to as ‘medical’ or ‘medicinal’ cannabis.
**Regulatory approved cannabis-based medicines:** those medicines routed through high-quality clinical trial programmes and approved for use by medicines regulators (e.g. the FDA, EMA or country-specific regulators).
EMA: European Medicines Agency, FDA: US Food and Drug Administration, HCP: healthcare professional, IQR: interquartile range

**Table S12** Patient questions about cannabis-based products

| **Question that survey participants (N = 737)^a^ were asked** | **Response** | **Respondents, n (%)** |
| --- | --- | --- |
| How do they ask about cannabis-based products? | They just ask about cannabis-based products in general,  they don't mention any names in particular | 735 (99.7) |
|  | They mention specific cannabis-based product names | 2 (0.3) |

^a^Restricted to respondents who answered yes to the question “Do your patients or their caregivers ever ask you about using any cannabis-based products for treating their condition?”

**Table S13** Knowledge of HCPs regarding cannabinoids and the cannabis plant

| **Question that survey participants (N = 1580) were asked** | **Response** | **Proportion of respondents, n (%)** |
| --- | --- | --- |
| Which, if any, of these cannabinoids are responsible for the intoxicating effects (‘high’) associated with recreational cannabis use? | THC alone | 1570 (99.4) |
|  | CBD alone | 2 (0.1) |
|  | Both THC and CBD | 8 (0.5) |
|  | Neither | 0 |
| Which parts of the cannabis plant produce the highest level of cannabinoids?^a^ | Seeds | 145 (9.2) |
|  | Flowers and upper leaves | 1517 (96.0) |
|  | Lower leaves | 6 (0.4) |
|  | Stem | 0 |
|  | Roots | 0 |
|  | I have no idea | 3 (0.2) |
|  | No Answer | 1 (0.1) |

^a^Respondents could select more than one response.

CBD: cannabidiol, HCP: healthcare professional, THC: delta-9-tetrahydrocannabinol

**Table S14** Confidence of respondents in the body of evidence for the safety and efficacy of cannabis-based products in healthcare based on their regulatory approval status

| **Question that survey participants (N = 1580) were asked** | **Scale** | **Median (IQR)** |
| --- | --- | --- |
| To what extent do you agree that there is a full and thorough body of evidence to support the **safety** of cannabis-based medicines that **have been approved by regulatory agencies**? | 1–5  (Completely disagree–completely agree) | 4 (4–4) |
| To what extent do you agree that there is a full and thorough body of evidence to support the **safety** of cannabis-based products for medical use that **have not been approved by regulatory agencies** (‘medical cannabis’)? |  | 3 (2–3) |
| To what extent do you agree that there is a full and thorough body of evidence to support the **efficacy** of cannabis-based medicines that **have been approved by medical regulatory agencies**? |  | 4 (4–4) |
| To what extent do you agree that there is a full and thorough body of evidence to support the **efficacy** of cannabis-based products for medical use that **have not been approved by regulatory agencies** (‘medical cannabis’)? |  | 3 (2–3) |

IQR: interquartile range

**Table S15** Confidence of respondents on the legality of cannabis-based products in healthcare

| **Question that survey participants (N = 1580) were asked** | | **Scale** | **Median (IQR)** |
| --- | --- | --- | --- |
| How confident are you right now that it is illegal/legal in your country for you to [prescribe/recommend/dispense]^a^ a cannabis-based product of these types? | Regulatory approved cannabis-based medicine | 1–5  (I am very confident it is illegal–It is definitely legal) | 5 (4–5) |
|  | Non-regulatory approved cannabis-based product |  | 3 (3–3) |

^a^Question wording differed according to the speciality of the respondent.

The below definitions of non-regulatory approved cannabis-based products and regulatory approved cannabis-based medicines were provided and made accessible to respondents while answering these questions:

**Non-regulatory approved cannabis-based products:** cannabis-based products used for medical purposes via prescription but not approved by medicines regulators – often referred to as ‘medical’ or ‘medicinal’ cannabis.

**Regulatory approved cannabis-based medicines:** those medicines routed through high-quality clinical trial programmes and approved for use by medicines regulators (e.g. the FDA, EMA or country-specific regulators).

EMA: European Medicines Agency, FDA: US Food and Drug Administration, IQR: interquartile range

**Table S16** Attitudes of HCPs towards the prescription or access of non-regulatory approved cannabis-based products

| **Question that survey participants (N = 1580) were asked** | **Response** | **Proportion of respondents, n (%)** |
| --- | --- | --- |
| Do you think you should be able to prescribe **non-regulatory approved** cannabis-based products to your patients?^a^  OR  Do you think patients should have access to  **non-regulatory approved** cannabis-based products?^b^ | Yes | 186 (11.8) |
|  | No | 234 (14.8) |
|  | Not sure | 1160 (73.4) |

^a^Neurologists, psychiatrists and general practitioners were asked this question.

^b^Pharmacists and nurses were asked this question.

The below definitions of non-regulatory approved cannabis-based products and regulatory approved cannabis-based medicines were provided and made accessible to respondents while answering these questions:

**Non-regulatory approved cannabis-based products:** cannabis-based products used for medical purposes via prescription but not approved by medicines regulators – often referred to as ‘medical’ or ‘medicinal’ cannabis.

**Regulatory approved cannabis-based medicines:** those medicines routed through high-quality clinical trial programmes and approved for use by medicines regulators (e.g. the FDA, EMA or country-specific regulators).

EMA: European Medicines Agency, FDA: US Food and Drug Administration, HCP: healthcare professional

**Table S17** Preferred method of receiving information on the legality, regulations, neurobiology, efficacy and safety, and clinical pharmacology of cannabis-based products

| **Question that survey participants (N = 1580)  were asked** | **Response** | **Respondents, n (%)** |
| --- | --- | --- |
| Through which sources would you prefer to receive information on legality, regulations, neurobiology, efficacy and safety, and clinical pharmacology of cannabis-based products? | Publications and scientific articles | 741 (46.9) |
|  | Scientific communication at congresses  (posters, presentations, symposia) | 303 (19.2) |
|  | Newsletters on cannabinoid science and conference updates | 122 (7.7) |
|  | Educational expert webinars provided by pharmaceutical company | 53 (3.4) |
|  | CME programmes from independent professional bodies | 51 (3.2) |
|  | Face-to-face visits from pharmaceutical company representatives | 47 (3.0) |
|  | Educational infographics provided by pharmaceutical company | 47 (3.0) |
|  | Telephone or video meetings with pharmaceutical  company representatives | 39 (2.5) |
|  | Educational slide decks provided by pharmaceutical company | 39 (2.5) |
|  | Patient-support materials provided by pharmaceutical company | 39 (2.5) |
|  | Educational podcasts provided by pharmaceutical company | 34 (2.2) |
|  | Peer-to-peer meetings organised by pharmaceutical company | 33 (2.1) |
|  | Educational animations and videos provided by  pharmaceutical company | 32 (2.0) |
|  | Other | 0 |

CME: continuing medical education

**Additional files**

**File name:** Additional file 1.

**File format:** .pdf

**Title of data:** Questionnaire provided to participants

**Description of data:** This document contains the survey questions that were answered by survey participants.

**File name:** Additional file 2

**File format:** .xls

**Title of data:** Raw data from survey responses.

**Description of data:** This spreadsheet contains the raw data necessary to interpret, replicate and build upon the findings reported in the article.
